# Enhancement of visual cortex plasticity by dark exposure

**DOI:** 10.1098/rstb.2016.0159

**Published:** 2017-03-05

**Authors:** Irina Erchova, Asta Vasalauskaite, Valentina Longo, Frank Sengpiel

**Affiliations:** School of Biosciences and Neuroscience and Mental Health Research Institute, Cardiff University, Sir Martin Evans Building, Museum Avenue, Cardiff, CF10 3AX, UK

**Keywords:** visual cortex, ocular dominance, mouse, plasticity, imaging, parvalbumin

## Abstract

Dark rearing is known to delay the time course of the critical period for ocular dominance plasticity in the visual cortex. Recent evidence suggests that a period of dark exposure (DE) may enhance or reinstate plasticity even after closure of the critical period, mediated through modification of the excitatory–inhibitory balance and/or removal of structural brakes on plasticity. Here, we investigated the effects of a week of DE on the recovery from a month of monocular deprivation (MD) in the primary visual cortex (V1) of juvenile mice. Optical imaging of intrinsic signals revealed that ocular dominance in V1 of mice that had received DE recovered slightly more quickly than of mice that had not, but the level of recovery after three weeks was similar in both groups. Two-photon calcium imaging showed no significant difference in the recovery of orientation selectivity of excitatory neurons between the two groups. Parvalbumin-positive (PV+) interneurons exhibited a smaller ocular dominance shift during MD but again no differences in subsequent recovery. The percentage of PV+ cells surrounded by perineuronal nets, a structural brake on plasticity, was lower in mice with than those without DE. Overall, DE causes a modest enhancement of mouse visual cortex plasticity.

This article is part of the themed issue ‘Integrating Hebbian and homeostatic plasticity’.

## 1. Background

The concept of a critical period for experience-dependent plasticity in the visual cortex was established through the seminal work of Hubel & Wiesel [1] on the effects of monocular deprivation (MD) in cat visual cortex. Dark rearing from birth was the first experiential manipulation that could be shown to affect the time course of the critical period, delaying its onset and prolonging its duration in cats, rats and mice [2–6]. In addition, studies of dark-reared cats and ferrets provided compelling evidence that key response properties of neurons in the primary visual cortex, such as orientation selectivity and ocular dominance are present at least in a significant number of cells in the absence of any visual experience [7,8]. However, prolonged dark rearing results in a progressive loss of neuronal selectivity and responsiveness, indicating that visual experience is necessary for their maintenance [9,10].

More recent work has elucidated several cellular and molecular effects of dark rearing. It was first shown in cats that dark rearing slows down the NMDA receptor-mediated component of visual responses in cortical layers 4, 5 and 6 [11]. Subsequent studies in rats showed that the NMDA receptor unit composition is bidirectionally modified by visual experience, with a progressive inclusion of GluN2A instead of GluN2B subunits during normal development. Light exposure following dark rearing triggers a rapid insertion of new NMDA receptors with a higher proportion of GluN2A than GluN2B subunits, while animals deprived of light exhibit a gradual switch in the opposite direction, from GluN2A to GluN2B subunits [12,13].

The effects of dark rearing on the time course of the critical period are twofold. On the one hand, dark rearing affects the excitatory–inhibitory balance by delaying the expression of brain-derived neurotrophic factor (BDNF) which in turn delays the maturation of intracortical inhibition [14]. The start of the critical period is thought to depend on a threshold level of inhibition being attained [15]. On the other hand, dark rearing also slows down the maturation of structural brakes on plasticity, such as the aggregation of chondroitin sulfate proteoglycans (CSPGs) and formation of perineuronal nets (PNNs), specifically around parvalbumin-positive (PV+) GABAergic basket cells [16] (for a review see [17]).

Finally, a non-Hebbian form of synaptic plasticity, which scales synaptic strengths up or down to stabilize average firing rates, has been demonstrated in rodent visual cortex. The decrease in amplitude of miniature excitatory postsynaptic currents (mEPSCs) in principal neurons which is normally observed between postnatal days 12 and 23 can be prevented by rearing rats in darkness from 12 days of age [18]. Moreover, dark exposure (DE) of normally raised mice showed that this experience-dependent homeostatic plasticity persists through adulthood. Two days of DE cause an increase in AMPA receptor-mediated mEPSC amplitude in the superficial layers of the visual cortex, which is rapidly reversed by one day of light exposure (LE) [19].

These findings raise the question whether a period of DE later in life might have an effect on visual cortical plasticity. Several studies in cats and rodents suggest that this is indeed the case. In adult rats that had been monocularly deprived from eye-opening, a period of 10 days of DE immediately prior to re-opening the deprived eye precipitated recovery of vision in the deprived eye as assessed by visual evoked potentials and a visual discrimination task [20]. Similarly, kittens that had been monocularly deprived for one week at the peak of the critical period showed only modest recovery of visual acuity when tested over two months after re-opening of the deprived eye; but after 10 days of DE acuity returned to normal levels within a few days [21]. Assessment of levels of neurofilament, another putative brake on plasticity, revealed a steady increase during normal development but a reduction following DE [21]. These studies again have added significance because they suggest a possible treatment for amblyopia in humans (for a review see [22]).

It is as yet unknown what the longer-term benefits of DE for recovery from MD (and amblyopia) are, and how responses of individual neurons change over time. Here, we employed chronic optical imaging of intrinsic signals and two-photon calcium imaging to compare rates and extent of recovery from MD with or without a brief period of DE. The density of PNNs and of PV+ GABAergic interneurons surrounded by such nets was assessed as a marker of structural plasticity.

## 2. Material and methods

Experiments were carried out on mice expressing the red fluorescent protein tdTomato in all PV+ cortical neurons. Mice were bred in-house by crossing PV^Cre^ mice (B6;129P2-*Pvalb^tm1 (cre)Arbr^*/J, JAX 008069, The Jackson Laboratory) with B6.Cg-*Gt(ROSA)26Sor^tm14(CAG-tdTomato)Hze^*/J mice (JAX 007914, The Jackson Laboratory). Both male and female offspring were used. Litter mates were randomly allocated to different experimental groups.

### (a) Monocular deprivation

Mice underwent MD by lid suture on postnatal day P26–P28. Anaesthesia was induced with 3% isoflurane in 100% oxygen and maintained at 1.5% isoflurane. An injection of Metacam (1 mg kg^−1^ s.c.) was given to alleviate post-operative pain. The lid margins of the left eye were trimmed and the lids sutured shut with two mattress sutures. A topical antibiotic ointment (chloramphenicol) was applied before animals were allowed to recover and were returned to the home cage. Animals were checked daily to ensure that the eye remained closed. All mice were monocularly deprived for 30 days. The deprived eye was re-opened in the beginning of the first optical imaging session (see below), and an ophthalmoscope was used to verify clarity of optic media.

### (b) Implantation of cranial window and virus injection

Two to three weeks before re-opening of the deprived eye cranial window surgery was performed. Anaesthesia was induced in an induction chamber with 3–4% isoflurane and 0.3 l min^−1^ oxygen. Mice were given Metacam (1 mg kg^−1^ s.c.) for analgesia and dexamethasone (2 mg kg^−1^ i.m.) to prevent cerebral oedema. Mice were placed on a heating pad to maintain body temperature at 37°C and placed in a stereotaxic frame.

During surgery, anaesthesia was maintained at 2–2.5% isoflurane with 0.3 l min^−1^ oxygen. Eyes were covered with protective ophthalmic cream. The surgical area was disinfected three times each with 70% ethanol and Betadine solution. The skin was removed and the edges of the incision were attached to the skull with tissue adhesive (Vetbond, 3M). A custom-made stainless steel headplate was attached to the skull with dental acrylic (C&B Metabond), leaving a circular area of 6 mm diameter exposed over the right visual cortex. A 2.5 × 2.5 mm square craniotomy was made using a dental drill.

V1 was transfected with the ultrasensitive calcium indicator GCaMP6s [23] using AAV-mediated gene transfer (University of Pennsylvania Vector Core); 100–200 nl (virus titre of 10^11–12^ genomes ml^−1^) were injected into binocular V1 (V1b) of the right hemisphere at a depth of 200–300 µm. V1b was defined using stereotaxic coordinates of 3.2–3.5 mm lateral from lambda. The injection was performed with a 5 µl syringe (Hamilton), using an UltraMicro Pump with Micro4 controller (WPI, USA). The syringe was adapted with RN compression fittings (Hamilton) in order to accommodate glass pipettes. The glass pipettes were pulled using a horizontal Sutter P-80 pipette puller to achieve a tip with long taper and small diameter of 1–2 µm; the tip was then broken off under a dissecting microscope to a diameter of 20–30 µm. After the injection, a small 2.5 × 2.5 mm square glass was glued to a 6 mm round cover-glass using UV curable adhesive (#61, Norland Optical Adhesives). The edges of the glass were secured onto the skull using tissue adhesive and dental acrylic. The mouse was then placed in a heated recovery chamber before being returned to the home cage.

### (c) Optical imaging

Intrinsic signal imaging was performed on V1 contralateral to the deprived eye. V1 was imaged under anaesthesia using 0.7–1% isoflurane in O_2_ at 0.3 l min^−1^, supplemented with 25 µg of chlorprothixene as previously described [24]. Computer-controlled shutters were used to present stimuli to one eye at a time. These were generated by VSG5 (Cambridge Research Systems) and consisted of a bar of 40° in length and 4° width drifting upwards at a rate of 0.125 Hz and a speed of 15° s^−1^, presented on a cathode ray tube screen positioned in front of the mouse at a distance of 14 cm. The stimulus was presented to each eye six times. The cortex was illuminated with red light (700 ± 10 nm interference filter). Images were acquired with an Imager 3001 (Optical Imaging Inc, Mountainside, NJ) using an Adimec 50-R64 camera. For each pixel in the imaged region, phase and amplitude of the optical signal at 0.125 Hz were calculated by Fast Fourier Transform [25]. Response magnitudes are presented as Δ*R*/*R* values where *R* is light reflected. The binocular zone of V1 was determined by thresholding the ipsilateral eye response map at 60% of the maximum pixel value. The average pixel value was then calculated within this area for both the ipsilateral and contralateral response maps. This provided an absolute measure of cortical response to contralateral and ipsilateral eye stimulation. In addition, an ocular dominance index (ODI) was calculated on a pixel-by-pixel basis according to the formula
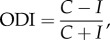
where *C* and *I* are the contralateral and ipsilateral eye response magnitudes, respectively. The overall ODI was taken as the median of the ODI values of all pixels in the binocular zone.

### (d) Two-photon calcium imaging

Mice underwent two-photon calcium imaging immediately after optical imaging as described above. Imaging was performed on a custom built 2P microscope (MOM, Sutter Instruments) equipped with Ti:Sapphire laser (MaiTai DeepSee, Newport SpectraPhysics) using a 20×, 1.00 NA Olympus (N20X-PFH) water immersion objective. A custom-build light protective cone was fitted around the objective to exclude extraneous illumination. The head of the animal was tilted 7°–16° to insure that the objective was parallel to the cortical surface. We used a digital inclinometer (Level Developments, UK) to determine the exact tilt and correct bar orientations accordingly (see below).

The laser was tuned to 940 nm and the power was maintained in the range of 25–35 mW. An area of 270 × 270 µm located in binocular V1 (V1b) was imaged at depths of 170–290 µm at 3.5 fps (ScanImage r3.6) while the mouse was presented with visual stimuli shown to one eye at a time. A 4° wide white bar on a grey background was presented at 16 directions of motion in 22.5° steps, drifting at 0.125 Hz. This was followed by 8 s of presentation of the grey background alone. The stimuli were presented four times to each eye for each area that was imaged. The red fluorescent tdTomato expressed in PV+ neurons was visualized at 1030 nm. After each imaging session mice were placed in a heated chamber until fully recovered and then returned to their home cage.

For analysis, we used custom written Matlab scripts (MathWorks). All images collected from a single area at the same resolution and wavelength were automatically aligned, and corrected for in-plane motion using a correlation-based subpixel registration. Images from the same conditions were averaged across the trials. Cell masks were manually assigned based on average and maximum calcium signals. Cellular fluorescence time courses were extracted by averaging the pixels within each cell mask and adjusted by subtracting the neuropil signal within a 20 µm spherical shell surrounding each cell. Visually evoked responses were computed as changes in fluorescence (Δ*F*/*F*) relative to the 2 s epoch prior to each stimulus for each cell. Maximum responses to each stimulus were used to construct orientation tuning curves.

We calculated an orientation selectivity index (OSI) by vector averaging [26] as OSI = 1 – circular variance (CV), with
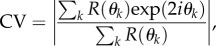
where *θ_k_* is the orientation of a visual stimulus and *R*(*θ_k_*) is the response to that stimulus.

The ODI of individual neurons was calculated as
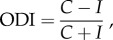


where *C* and *I* represent maximum contralateral and ipsilateral eye response magnitudes, respectively.

### (e) Immunohistochemistry, microscopy and analysis

At the end of the *in vivo* imaging experiments, animals were killed by cervical dislocation. The brains of mice that had not been transfected with GCaMP6s were dissected, fixed in 4% paraformaldehyde (PFA), frozen in isopentane and cut into coronal sections at 14 µm using a cryostat (CM 3050S, Leica) at −20°C. The frozen sections were air-dried for at least 30 min and then rehydrated with 0.1 M phosphate-buffered saline (PBS) two times for 5 min each.

For visualization of PNNs, the primary marker, Wisteria floribunda agglutinin (WFA) was applied (1 : 100 in PBS) and incubated overnight at 4°C. Sections were rinsed with PBS three times (5 min each) and the secondary antibody, Avidin-FITC was applied (1 : 500 in PBS) for 1 h at RT. After another three washes, sections were incubated in the nuclear stain Hoechst in dH_2_O for 5 min. Sections were cover-slipped with mounting media and stored at 4°C.

Sections were viewed using an epifluorescence microscope (DM6000, Leica). Appropriate filters were used for visualizing WFA staining (green: PNNs, FITC) and tdTomato fluorescence (red: PV-expressing cells). Typically, four sections (1 in 2 sections collected) were captured per animal and hemisphere.

All images were analysed using Fiji/ImageJ software [27]. A 500 × 500 µm region of interest (ROI) was analysed per image, positioned below layer 1 of V1. PNNs and PV+ cells were counted separately per image. Then both images were superimposed and cells showing colocalization were counted. The cell counter plugin (Fiji) was used for recording the counts. The percentages of PV+ cells surrounded by PNNs (% colocalization) were calculated as:
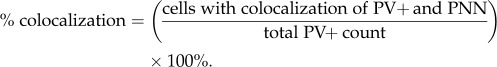


### (f) Parvalbumin-positive cell counts *in vivo*

In some mice, we analysed the density of PV+ cells in image stacks obtained with two-photon imaging. For this purpose, 512 × 512 pixel images were taken every 5 µm at 20× magnification; 80–100 images were taken starting at the dura. For each image in the stack, cells within a 3000 pixel^2^ grid (with a pixel being 0.64 µm^2^) were counted, and cell density per cubic millimetre was calculated.

### (g) Statistical analysis

Data are reported as mean ± standard error of the mean (s.e.m.), unless otherwise noted. Statistical analyses were performed with Prism GraphPad (v. 7) and SPSS (v. 20) software. Normality of distribution was tested and parametric ANOVA for comparisons between the experimental groups and time points was employed followed by multiple comparison test (Holm-Sidak).

## 3. Results

### (a) Intrinsic signal imaging

**Figure 1.**
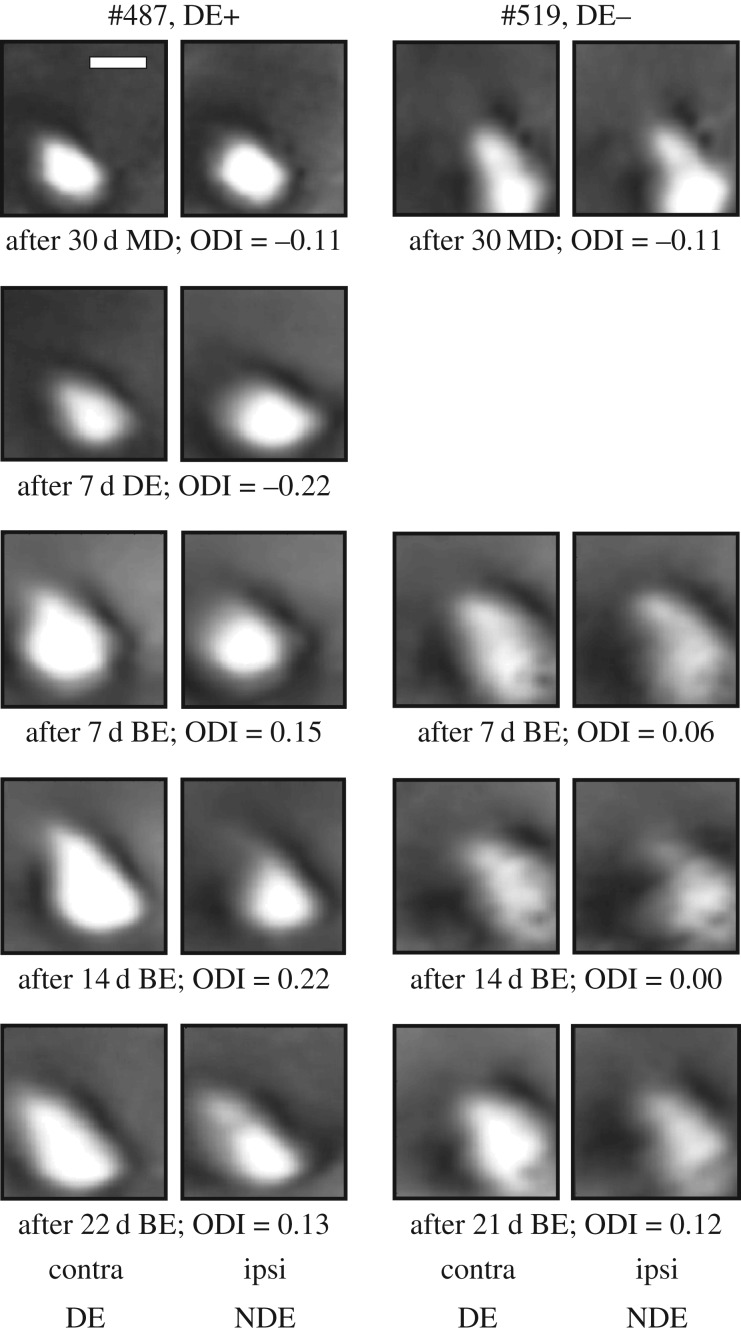
Recovery of V1 responses following 30 d MD in a mouse that experienced a week of dark exposure before binocular exposure (left two columns, #487, DE+) and a mouse that received binocular exposure (BE) immediately after MD (right two columns, #519, DE−). Magnitude maps of intrinsic signals for left, deprived-eye (contra DE) and right, non-deprived eye (ipsi NDE) stimulation are shown for different time points after MD; ODI values are given below each pair of response maps. Scale bar, 1 mm.

Longitudinal intrinsic signal imaging data were obtained from 12 mice in which binocular exposure (BE) immediately followed long-term MD (DE− group) and nine mice which experienced 7 days of DE before the BE (DE+ group). Examples of activity maps elicited through left- and right-eye stimulation immediately after the end of the MD period and then at weekly intervals during the recovery period are shown in figure 1 for one animal from the DE− group and one animal from the DE+ group. Both animals exhibit a strong increase in response to stimulation of the previously deprived eye during the first week of BE and comparatively smaller changes in response through either eye thereafter.

**Figure 2.**
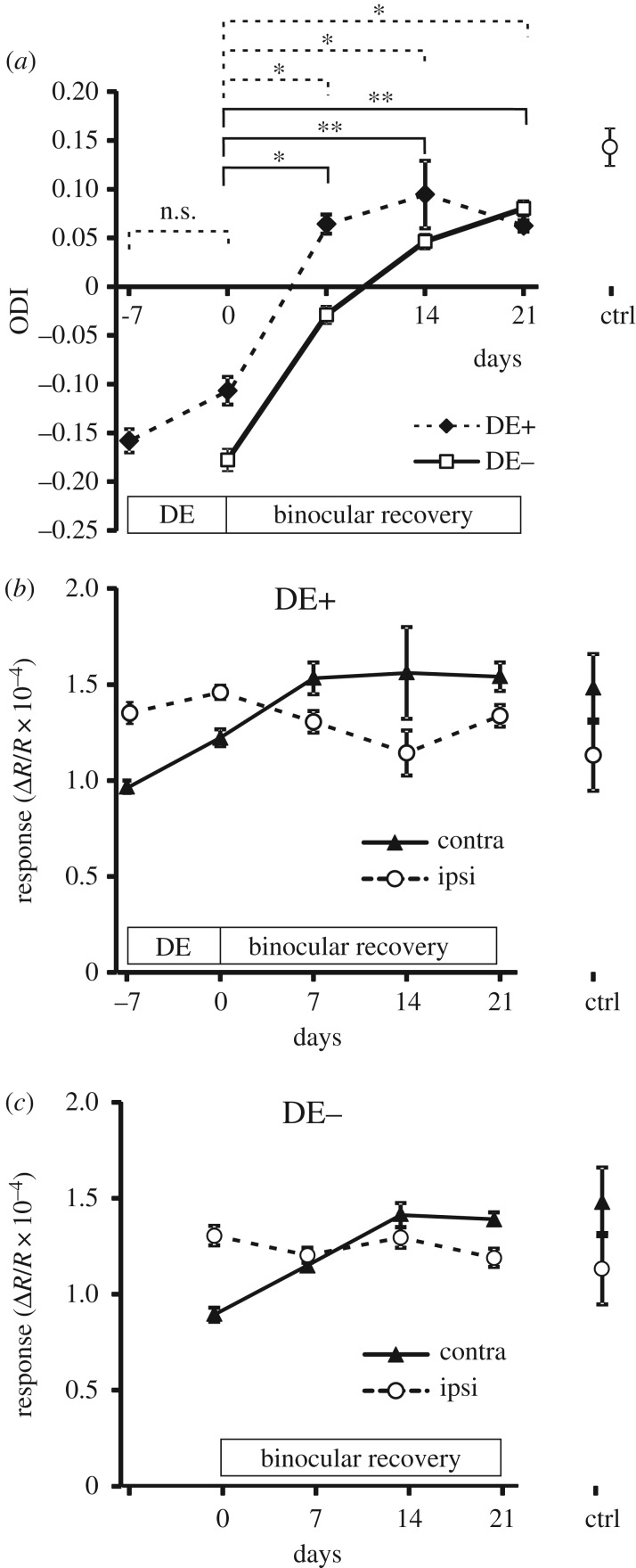
Time course of ODI and of left- and right-eye response magnitudes obtained from chronic intrinsic signal imaging of mice with (DE+) and without a week of dark exposure (DE−) following MD. (*a*) Changes in ODI for DE+ (filled circles, dotted lines) and DE− cohorts (open squares, solid lines). Significant changes are indicated for the DE+ group (dotted line brackets) and DE− group (solid line brackets); n.s., non-significant; *p < 0.05; **p < 0.01 (Holm-Sidak multiple comparisons test). (*b*) Magnitude of responses through the contralateral previously deprived eye (filled triangles, solid lines) and the ipsilateral eye (open circles, dashed lines) in the DE+ cohort. (*c*) Magnitude of contra- and ipsilateral eye responses in the DE− cohort.

After averaging the ODI at all time points across all animals in both groups (figure 2*a*), it is again apparent that the largest OD shift occurs within the first week of BE. This shift, when compared with the ODI at the end of the MD period (−0.16 ± 0.01 in the DE+ group and −0.18 ± 0.01 in the DE− group, both mean ± s.e.m.), is greater in the group that experienced a week of DE (ODI, 0.064 ± 0.010) than in the group that did not (ODI, −0.029 ± 0.009); however the difference between the groups is not significant (*p* = 0.087). Moreover, the magnitude of the OD shift during the first 7 days of BE is only marginally greater in the DE+ group (ΔODI = 0.180) compared with the DE− group (ΔODI = 0.155). After a total three weeks of binocular experience, the ODI values were virtually identical for the two groups, 0.062 ± 0.006 for the DE+ group and 0.080 ± 0.008 for the DE− group. The difference in recovery between the DE+ and DE− groups therefore appears to be primarily due to the OD shift in the DE+ group during the 7 days of DE which is however not significant (*p* > 0.1). In order to assess the effect of DE alone on ocular dominance, we additionally imaged three groups of mice which were dark-reared from birth for eight weeks (*n* = 9), dark exposed from P20 for eight weeks (*n* = 7), or dark exposed from P60 for eight weeks (*n* = 5). DE resulted in a significant (*p* < 0.01) reduction in ODI towards zero in all three groups compared with normally reared control animals, and this reduction was greater the earlier DE had started (electronic supplementary material, figure 1*a*). DE caused a reduction in responsiveness through both eyes but more so through the contralateral eye (electronic supplementary material, figure 1*b*), resulting in more balanced responses through the two eyes. By contrast, in the DE+ group a small increase in response through the deprived (contralateral) eye was observed following DE (figure 2*b*).

Closer inspection of the magnitude of the responses evoked through each eye reveals that in both groups the observed recovery is accounted for by an increase in response to stimulation of the contralateral, previously deprived eye, while the magnitude of responses through the ipsilateral eye remained substantially unchanged throughout the observation period (figure 2*b*,*c*). This increase in deprived-eye responses occurs during the first two weeks after the end of MD.

### (b) Calcium imaging

We carried out calcium imaging on 13 animals that had undergone MD, six of which received BE immediately (DE−) and seven received a week of DE prior to binocular recovery (DE+). All of these animals were imaged at weekly intervals, and two to four areas were imaged per session. In addition, we imaged seven age-matched control animals (three of which were littermates of experimental subjects), in order to assess normal visual responses in terms of ocular dominance and orientation selectivity.

**Figure 3.**
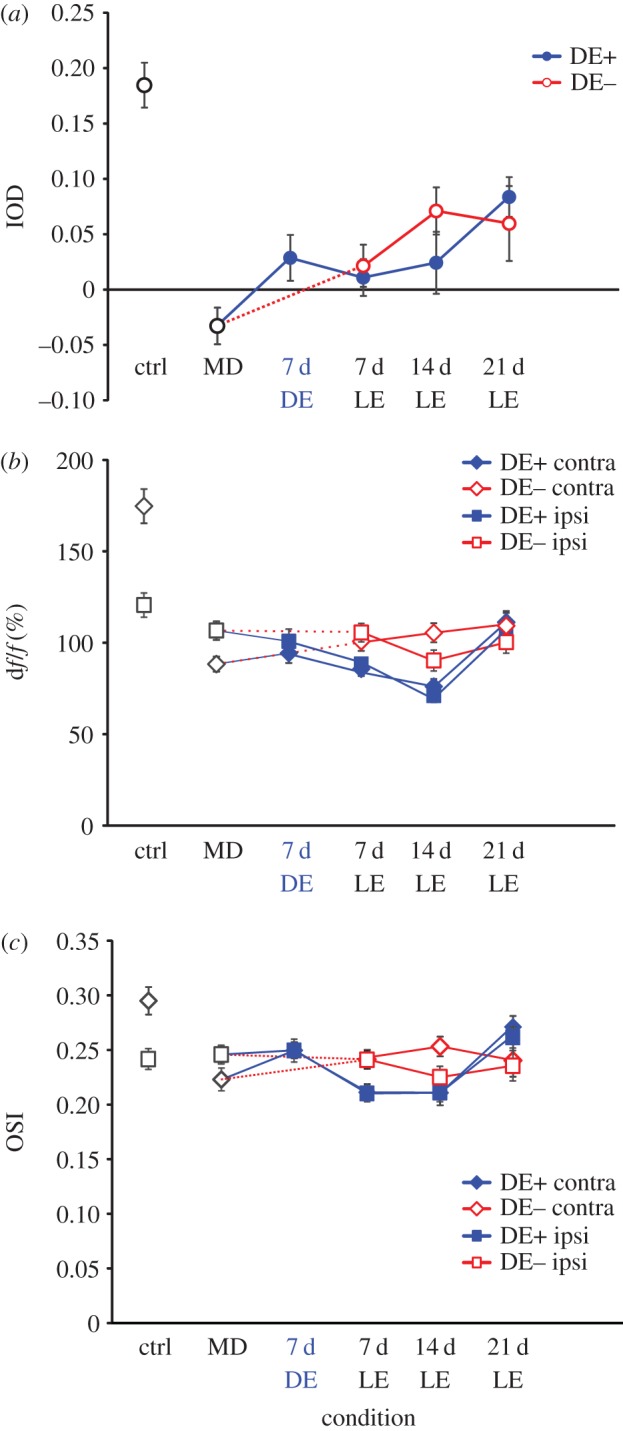
Population responses of excitatory neurons in control animals, in monocularly deprived mice, and during recovery from MD (LE, light exposure). (*a*) Ocular dominance index; open red circles represent data from DE− mice, filled blue circles represent data from DE+ mice. (*b*) Magnitude of neuronal responses through the contralateral eye (diamonds) and the ipsilateral eye (squares) in control (ctrl, black symbols), DE+ (filled blue symbols) and DE− mice (open red symbols). (*c*) Orientation selectivity index of neuronal responses through the contralateral eye (diamonds) and the ipsilateral eye (squares) in control, DE+ and DE− mice.

Excitatory neurons exhibited the expected ODI shift, from 0.185 ± 0.017 in normally reared control animals (*n* = 161 cells) to −0.033 ±0.015 (*n* = 268 cells) in the experimental animals at the end of the 30 d MD period. Compared with the optical imaging signals, the recovery single-cell responses through the previously deprived eye were slower and less complete (figure 3). Following three weeks of binocular recovery, the ODI increased to 0.054 ± 0.015 (*n* = 235 cells) in the DE− group and to 0.084 ± 0.015 (*n* = 215 cells) in the DE+ group (figure 3*a*); there was however no significant difference between the DE+ and DE− groups (*p* > 0.1). Similarly, the contralateral (previously deprived) eye response was drastically reduced immediately after 30 d MD compared with normally reared control animals (figure 3*b*) and increased only gradually and incompletely during the subsequent binocular recovery period. At the same time, the ipsilateral eye responses remained largely unchanged in both the DE+ and DE− groups. Orientation selectivity of contralateral eye responses was reduced significantly, from 0.295 ± 0.013 to 0.223 ± 0.007 (*p* < 0.001, 2-tailed *t*-test) after 30 d MD compared with control animals (figure 3*c*), and did not recover substantially during the BE period in both DE+ and DE− groups, while the OSI for ipsilateral (open) eye responses remained stable across rearing conditions and time.

**Figure 4.**
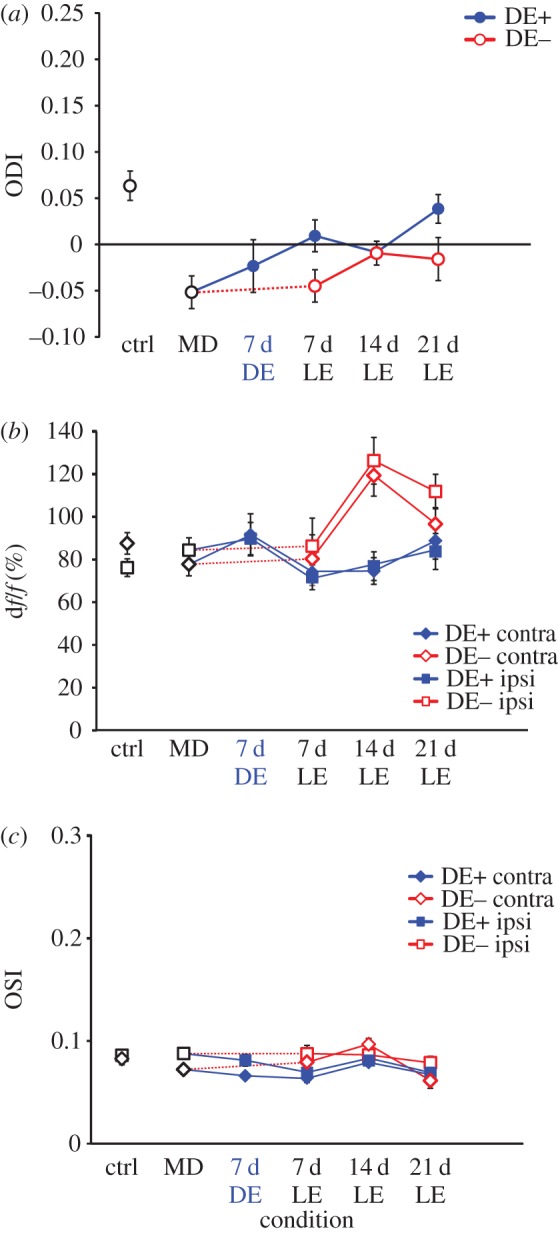
Population responses of PV+ inhibitory neurons in control animals, in monocularly deprived mice, and during recovery from MD. (*a*) Ocular dominance index; open red circles represent data from DE− mice, filled blue circles represent data from DE+ mice. (*b*) Magnitude of neuronal responses through the contralateral eye (diamonds) and the ipsilateral eye (squares) in control, DE+ and DE− mice. (*c*) Orientation selectivity index of neuronal responses through the contralateral eye (diamonds) and the ipsilateral eye (squares) in control, DE+ and DE− mice.

PV+ inhibitory neurons, identified by their red fluorescence, exhibited a much weaker contralateral bias in normally reared mice (0.063 ± 0.016, *n* = 86 cells) and a weaker OD shift towards the ipsilateral eye after 30 d MD (ODI = −0.052 ± 0.018, *n* = 130 cells). An example of the identification of individual PV+ cells across imaging sessions and typical responses are shown in electronic supplementary material, figure S2. With or without an intervening period of DE the ODI then recovered towards the original value during subsequent BE (figure 4). Analysis of responses through each eye revealed that contralateral eye responses decreased slightly in MD compared with control animals while ipsilateral eye responses increased slightly. There was no consistent trend in the amplitude of individual eye responses during the binocular recovery period. As expected, orientation selectivity of PV+ cell responses was very low in normally reared control animals (OSI = 0.083 ± 0.006 for contralateral eye stimulation) and did not change either during MD (OSI = 0.072 ± 0.004 for contralateral eye stimulation) or subsequent recovery.

### (c) Parvalbumin-positive cells and perineuronal nets

**Figure 5.**
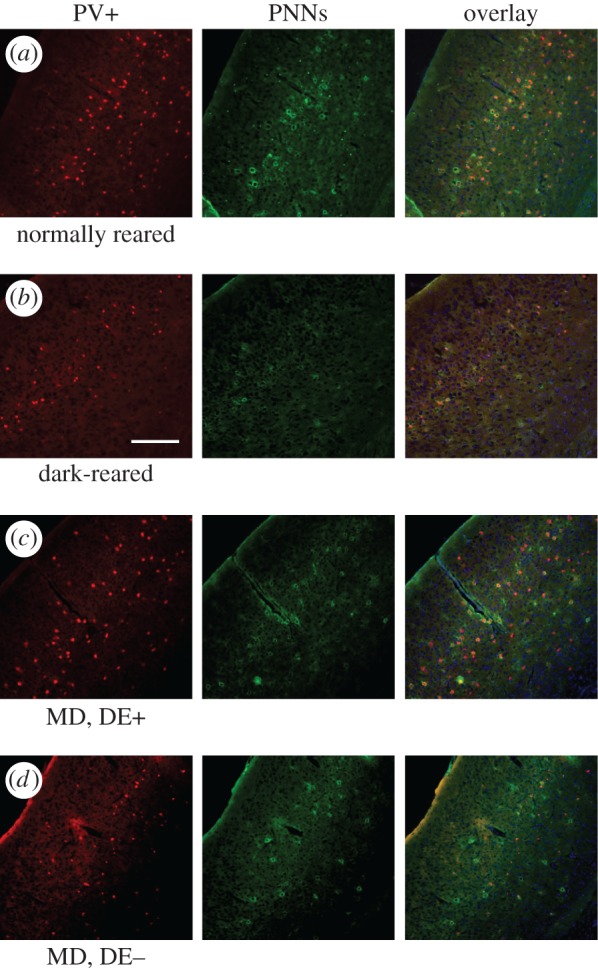
Fluorescence photomicrographs of coronal V1 sections from four mice reared with different visual experiences. (*a*) Normally reared mouse aged eight weeks. (*b*) Dark-reared mouse aged eight weeks. (*c*) Mouse with 30 d MD followed by one week of dark exposure and subsequent binocular recovery. (*d*) Mouse with 30 d MD followed immediately by four weeks of binocular recovery. Scale bar, 200 µm.

**Figure 6.**
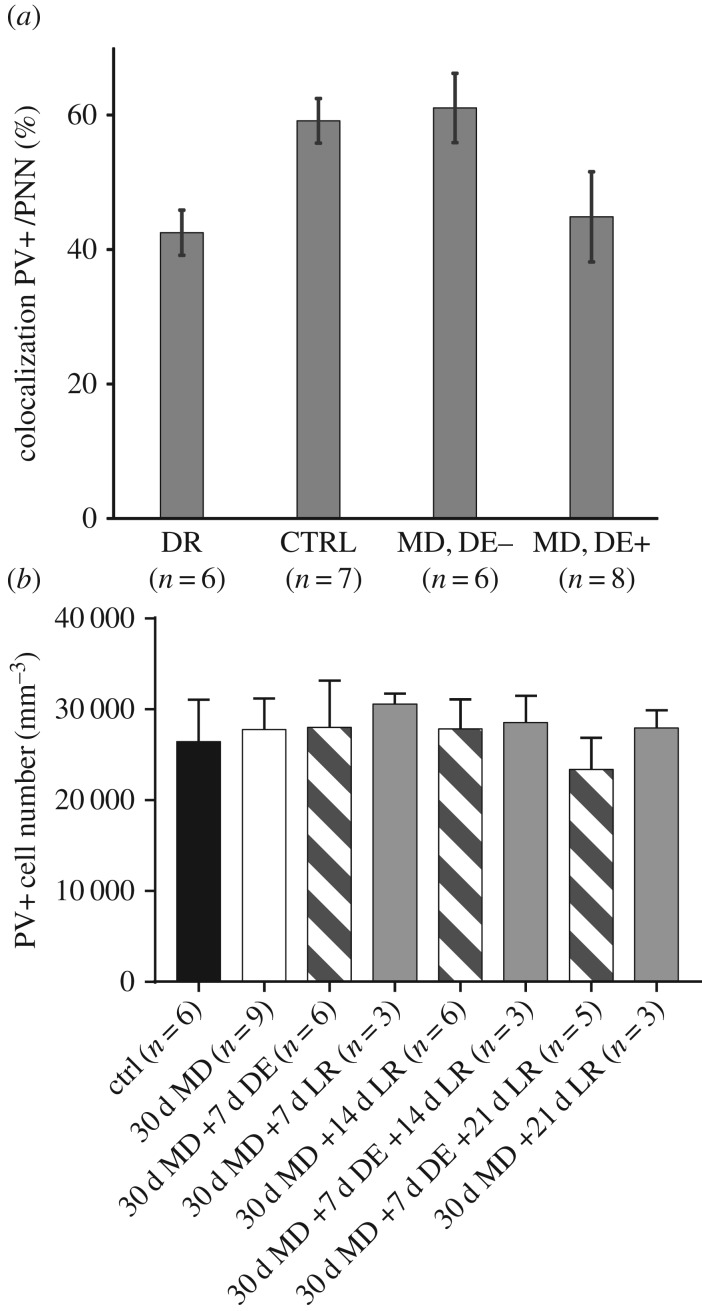
Density of perineuronal nets and of PV+ cells in different rearing conditions. (*a*) Percentage of PV+ cells that are surrounded by PNNs are plotted against rearing condition. Values are significantly higher in the normally reared (CTRL) and in the MD animals without subsequent dark exposure (MD, DE−) than in the dark-reared animal (DE) and the MD mice that had a week of dark exposure after re-opening of the deprived eye (MD, DE+). (*b*) Density of PV+ cells for all rearing conditions, in image stacks obtained *in vivo* by two-photon imaging. No significant differences were observed between any of the conditions.

Cell counts were performed on sections collected from eight DE+ mice and six DE− mice. Additionally, seven age-matched normally reared mice and six mice dark-reared from birth served as control groups. Figure 5 shows representative examples of results obtained from one animal in each of the four groups. With respect to the density of PV+ cells and perineuronal nets (PNNs), there were no significant differences between the two cortical hemispheres in any condition; these were therefore combined. By contrast, rearing condition had a significant effect on the percentage of PV+ cells that were surrounded by PNNs. This fraction was 59.13% ± 3.31% in normally reared mice but only 42.50% ± 3.35% (mean ± s.e.m.) in dark-reared mice (figure 6*a*); this was significantly less than normal and in line with previous reports that dark-rearing delays the maturation of PNNs [16].

In the mice that had received a week of DE following MD (DE+) the fraction of PV+ cells with PNNs was 44.86 ± 6.70%. This fraction was significantly lower (*t* test, *p* < 0.05) than in normally reared mice but similar to the value for the dark-reared group. By contrast, for mice in which BE immediately followed MD (DE−), the proportion of PV cells with PNNs was 61.05 ± 5.16%. This value was significantly higher than for both the DE+ group (*t* test, *p* < 0.05) and the dark-reared group (*t* test, *p* < 0.001) but similar to the normally reared control group.

Furthermore, absolute PV+ cell numbers were counted *in vivo*, in image stacks obtained by two-photon imaging, from six normally reared mice, six DE+ and three DE− animals. Of the normally reared animals, four were imaged just once, the remaining two and all the DE+ and DE− animals were imaged three or four times at weekly intervals. In the normally reared control group, the PV+ cell density was 26 584 ± 1697 cells mm^−3^, in the DE+ group it was 26 713 ± 1098 cells mm^−3^, and in the DE− group it was 29 103 ± 547 cells mm^−3^ (figure 6*b*). PV+ cell density neither changed significantly over time in those animals that were imaged repeatedly, nor did it differ between rearing conditions at any time point (Dunn's multiple comparisons, all *p* > 0.1).

## 4. Discussion

We found that a one-week period of DE provides but a modest enhancement to the recovery from MD in young adult mice. The principal effects were (i) an accelerated recovery of deprived-eye responses as observed with intrinsic signal imaging, and (ii) a reduction in the proportion of PV+ cells surrounded by PNNs. There was also a (non-significant) trend for recovery of single-cell responses after DE to be more complete than without DE.

Taken together, DE appears to boost visual cortex plasticity in mice by less than it does in rats [20] and cats [21]. The most likely explanation for this species difference, however, is not that DE is ineffective in mice but that residual plasticity beyond the end of the classical critical period is much greater in mice than in rats and cats, such that simply re-opening the deprived eye and giving binocular visual exposure allows significant recovery in young adult mice but much less in rats [20] or cats [21]. In previous studies, this plasticity beyond the classical critical period (lasting from P19 to P32 [28]) has typically been manifest as an OD shift in response to MD which is mediated by an LTP-like potentiation of the non-deprived eye [29,30] as opposed to an LTD-type depression of deprived-eye responses (for a review see [31]). For C57BL/6 mice reared in standard cages, this extended OD plasticity can be observed until P110 [32] but for mice reared in much larger cages providing an enriched environment it can still be seen at seven months of age or more [33]. Interestingly, DE on its own causes a shift in ocular dominance towards a more balanced representation of the two eyes. This has not been reported before, but our results are consistent with an earlier study that found that dark-rearing from birth reduced visual responses while also shifting spine dynamics and morphologies toward an immature state [34].

Here, we show that recovery from MD which started during the critical period is mediated by potentiation of responses through the previously deprived eye in young adult mice. This contrasts with an earlier study in which adult (more than P120) and old mice (more than P500) were monocularly deprived for 7 days immediately *after* a 10–14 day period of DE; in these animals the observed OD shift was mediated by an increase in non-deprived-eye responses [35]. Notwithstanding the different sequences of MD and DE, it is most likely that the differences in eye-specific changes reflect mechanistic differences between the response to MD itself and the recovery from MD [31]. This hypothesis is based on the fact that the same sequence as employed here (DE after MD) did cause a very pronounced enhancement of ocular dominance plasticity in both rats [20] and kittens [21]. The most parsimonious explanation of the different eye-specific effects in our study and that by Stodieck and co-workers [35] is that in each case the original eye-specific MD effect was reversed—which in the case of young mice is deprived-eye depression but in older mice is potentiation of the non-deprived eye.

An important role for GABAergic inhibition in regulating ocular dominance plasticity was first shown by Hensch and co-workers [36] for mice in which an isoform of the GABA synthesizing enzyme glutamic acid decarboxylase (GAD) had been knocked out. Of the numerous classes of GABAergic interneurons in the visual cortex, the PV+ basket cells have been identified as key players [17], and the formation of PNNs around them has been shown to parallel the closure of the critical period [16]. However, there has been controversy over how inhibitory neurons respond to MD. Gandhi *et al.* [37] reported that the entire population of GABAergic interneurons displays a similar degree of contralateral eye dominance in non-deprived mice as excitatory neurons, a slower shift in OD following MD but eventually the same magnitude of OD shift. By contrast, Kameyama *et al.* [38] found that GABAergic neurons are slightly more binocular than excitatory neurons and exhibit a great OD shift when MD is imposed after the critical period. Our analysis was restricted to PV+ cells, which constitute about 40% of all GABAergic cells in V1 [39]. These were significantly more binocular than all the remaining visually responsive neurons, and their OD shift after 30 days of MD was weaker. Neither the deprived nor the open eye responses changed significantly. Saiepour and co-workers [40] recently showed that PV+ cells are the primary source of inhibition in mouse V1, and inhibitory and excitatory inputs to individual V1 neurons are normally balanced, but MD causes their relative strengths to diverge. Our results support their conclusion that OD plasticity is not caused by eye-specific changes in inhibition [40].

Our finding of a reduction in the proportion of PV+ cells surrounded by PNNs suggests that structural brakes to plasticity are loosened by DE. This is in agreement with the observation in cats that DE reduces neurofilament expression [21]. Stodieck *et al.* [35] also reported a decrease in the density of PNNs following DE in older mice. In addition, they found a reduction in the density of PV+ cells using immunofluorescence while in our study, using *in vivo* imaging of a genetically encoded fluorescent PV+ cell marker, we did not observe any differences in numbers of PV+ cells across rearing conditions. This discrepancy may be attributable to differences in the experimental paradigms or the method of analysis.

In summary, our data provide some evidence for the capacity of a brief period of DE to enhance plasticity in the primary visual cortex both in functional and structural terms.

## Supplementary Material

Effects of dark exposure alone on ocular dominance and individual eye response amplitude in mouse V1.

## Supplementary Material

Identification of individual PV+ cells across imaging sessions and visual responses to oriented gratings in mouse V1.

## Data accessibility

All original data and Matlab analysis routines are available from the corresponding author upon request. Electronic supplementary material, two figures are available online.
