# Supplementary figures and images for: Enhancement of visual cortex plasticity by dark exposure
Source: Philos Trans R Soc Lond B Biol Sci. 2017 Mar 5;372(1715):20160159. doi: 10.1098/rstb.2016.0159 (PMC5247591; doi:10.1098/rstb.2016.0159)

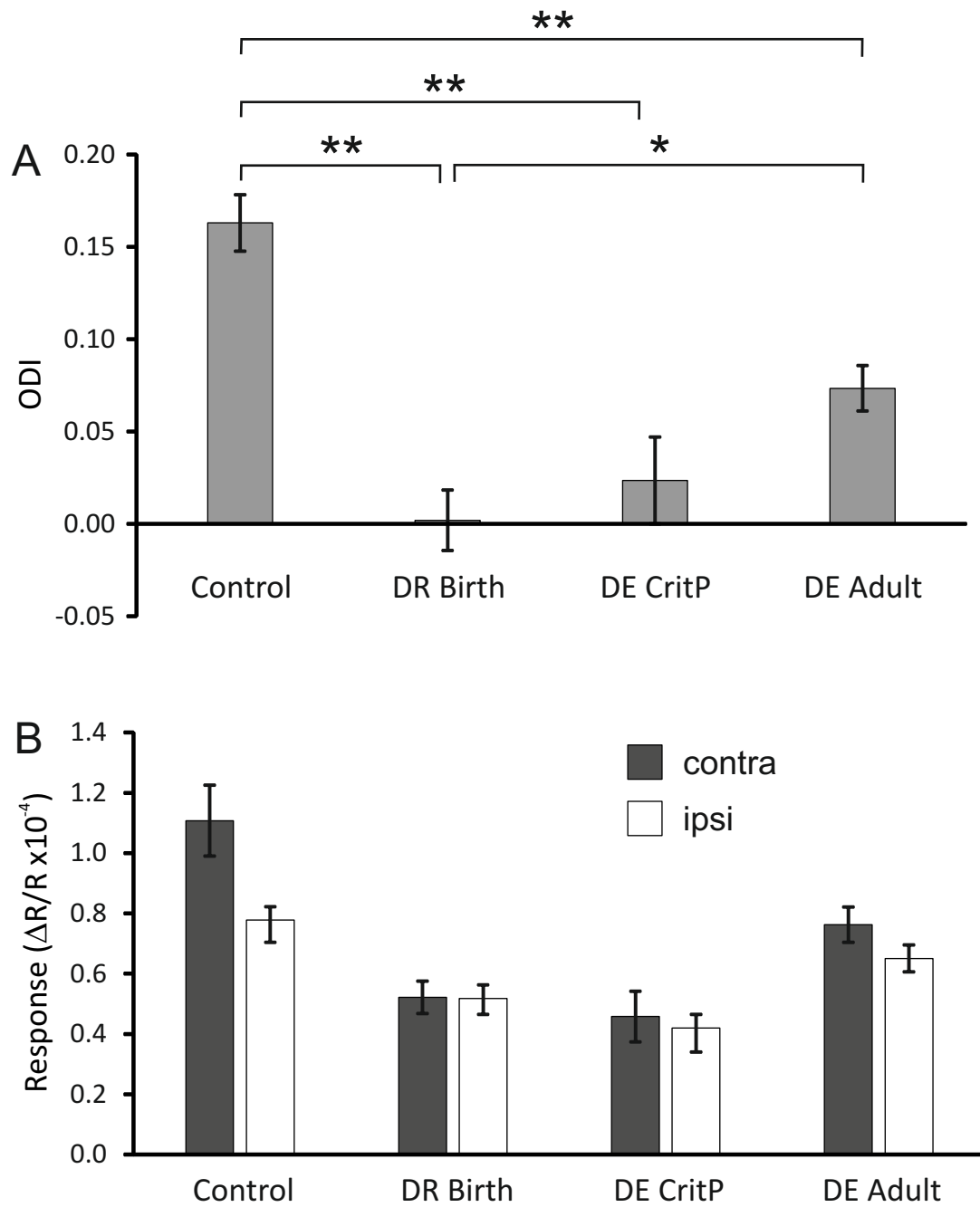

Supplementary Figure 1

Supplement: Effects of dark exposure alone on ocular dominance and individual eye response amplitude in mouse V1. [file rstb20160159supp1.pdf]

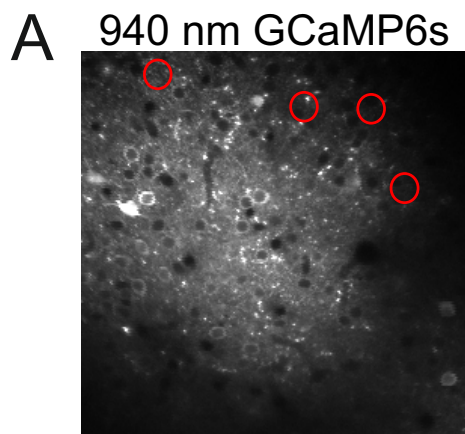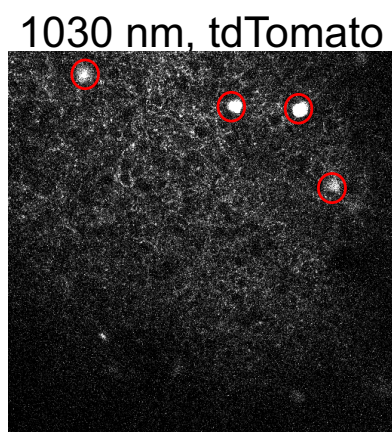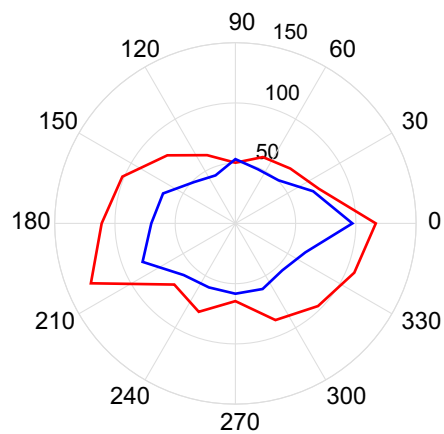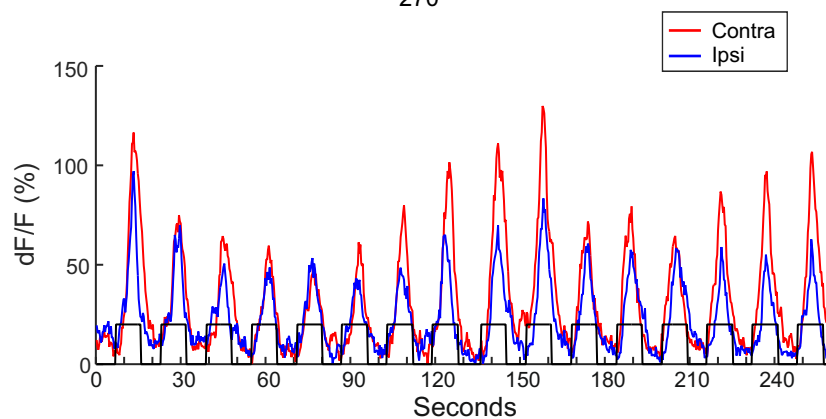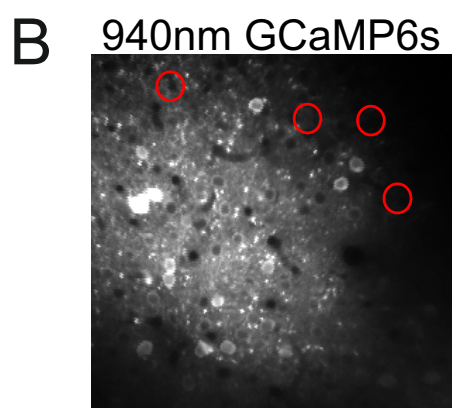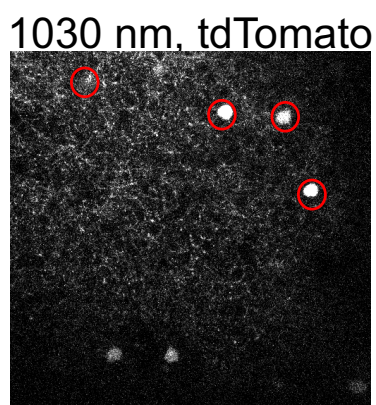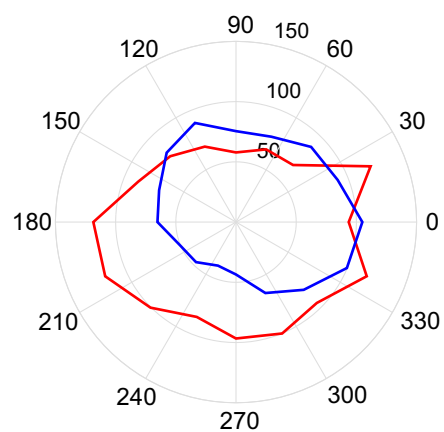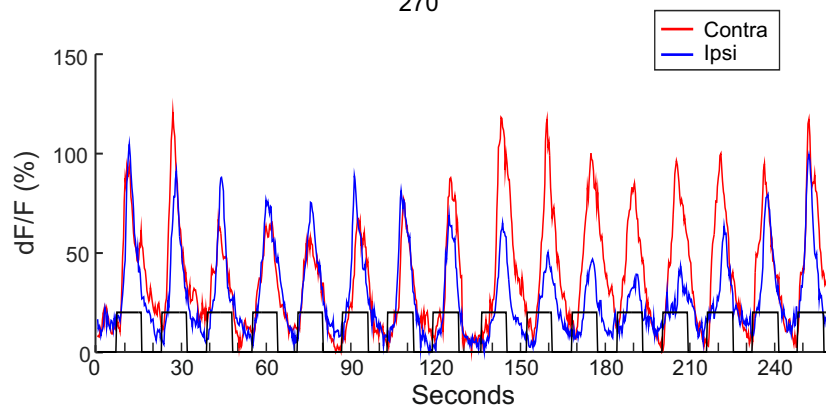

Supplementary Figure 2

Supplement: Identification of individual PV+ cells across imaging sessions and visual responses to oriented gratings in mouse V1. [file rstb20160159supp2.pdf]
